# Search for the in-situ production of $^{77}$Ge in the GERDA neutrinoless double-beta decay experiment

**DOI:** 10.1140/epjc/s10052-025-14445-x

**Published:** 2025-07-26

**Authors:** GERDA collaboration, M. Agostini, A. Alexander, G. Araujo, A. M. Bakalyarov, M. Balata, I. Barabanov, L. Baudis, C. Bauer, S. Belogurov, A. Bettini, L. Bezrukov, V. Biancacci, E. Bossio, V. Bothe, R. Brugnera, A. Caldwell, S. Calgaro, C. Cattadori, A. Chernogorov, P.-J. Chiu, T. Comellato, V. D’Andrea, E. V. Demidova, N. Di Marco, E. Doroshkevich, M. Fomina, A. Gangapshev, A. Garfagnini, C. Gooch, P. Grabmayr, V. Gurentsov, K. Gusev, J. Hakenmüller, S. Hemmer, W. Hofmann, J. Huang, M. Hult, L. V. Inzhechik, J. Janicskó Csáthy, J. Jochum, M. Junker, V. Kazalov, Y. Kermaïdic, H. Khushbakht, T. Kihm, K. Kilgus, I. V. Kirpichnikov, A. Klimenko, K. T. Knöpfle, O. Kochetov, V. N. Kornoukhov, P. Krause, V. V. Kuzminov, M. Laubenstein, M. Lindner, I. Lippi, A. Lubashevskiy, B. Lubsandorzhiev, G. Lutter, C. Macolino, B. Majorovits, W. Maneschg, G. Marshall, M. Misiaszek, M. Morella, Y. Müller, I. Nemchenok, M. Neuberger, L. Pandola, K. Pelczar, L. Pertoldi, P. Piseri, A. Pullia, C. Ransom, L. Rauscher, M. Redchuk, S. Riboldi, N. Rumyantseva, C. Sada, S. Sailer, F. Salamida, S. Schönert, J. Schreiner, A.-K. Schütz, O. Schulz, M. Schwarz, B. Schwingenheuer, O. Selivanenko, E. Shevchik, M. Shirchenko, L. Shtembari, H. Simgen, A. Smolnikov, D. Stukov, S. Sullivan, A. A. Vasenko, A. Veresnikova, C. Vignoli, K. von Sturm, T. Wester, C. Wiesinger, M. Wojcik, E. Yanovich, B. Zatschler, I. Zhitnikov, S. V. Zhukov, D. Zinatulina, A. Zschocke, K. Zuber, G. Zuzel

**Affiliations:** 1 INFN Laboratori Nazionali del Gran Sasso, Assergi, Italy; 2 INFN Laboratori Nazionali del Gran Sasso and Gran Sasso Science Institute, Assergi, Italy; 3 INFN Laboratori Nazionali del Gran Sasso and Università degli Studi dell’Aquila, L’Aquila, Italy; 4 INFN Laboratori Nazionali del Sud, Catania, Italy; 5 Institute of Physics, Jagiellonian University, Cracow, Poland; 6 Institut für Kern- und Teilchenphysik, Technische Universität Dresden, Dresden, Germany; 7 Joint Institute for Nuclear Research, Dubna, Russia; 8 European Commission, JRC-Geel, Geel, Belgium; 9 Max-Planck-Institut für Kernphysik, Heidelberg, Germany; 10 Department of Physics and Astronomy, University College London, London, UK; 11 INFN Milano Bicocca, Milan, Italy; 12 Dipartimento di Fisica, Università degli Studi di Milano, INFN Milano, Milan, Italy; 13 Institute for Nuclear Research, Russian Academy of Sciences, Moscow, Russia; 14 Institute for Theoretical and Experimental Physics, NRC “Kurchatov Institute”, Moscow, Russia; 15 National Research Centre “Kurchatov Institute”, Moscow, Russia; 16 Max-Planck-Institut für Physik, Munich, Germany; 17 Physik Department, Technische Universität München, Munich, Germany; 18 Dipartimento di Fisica e Astronomia, Università degli Studi di Padova, Padua, Italy; 19 INFN Padova, Padua, Italy; 20 Physikalisches Institut, Eberhard Karls Universität Tübingen, Tübingen, Germany; 21 Physik-Institut, Universität Zürich, Zurich, Switzerland; 22 NRNU MEPhI, Moscow, Russia; 23 Duke University, Durham, NC USA; 24 Moscow Inst. of Physics and Technology, Moscow, Russia; 25 Semilab Zrt, Budapest, Hungary; 26 Dubna State University, Dubna, Russia; 27 Nuclear Science Division, Berkeley, USA

## Abstract

The beta decay of $^{77}$Ge and $^{77\textrm{m}}$Ge, both produced by neutron capture on $^{76}$Ge, is a potential background for Germanium based neutrinoless double-beta decay search experiments such as GERDA or the LEGEND experiment. In this work we present a search for $^{77}$Ge decays in the full GERDA Phase II data set. A delayed coincidence method was employed to identify the decay of $^{77}$Ge via the isomeric state of $^{77}$As ($9/2^+$, ${475}\,\hbox {keV}$, ${T_{1/2} = {114}\,{\upmu }\hbox {s}}$, $^{77\textrm{m}}$As). New digital signal processing methods were employed to select and analyze pile-up signals. No signal was observed, and an upper limit on the production rate of $^{77}$Ge was set at $<0.216$ nuc/(kg$\cdot$ yr) (90% CL). This corresponds to a total production rate of $^{77}$Ge and $^{77\textrm{m}}$Ge of $<{0.38}$ nuc/(kg$\cdot$ yr) (90% CL), assuming equal production rates. A previous Monte Carlo study predicted a value for in-situ $^{77}$Ge and $^{77\textrm{m}}$Ge production of (0.21 ± 0.07) nuc/(kg.yr), a prediction that is now further corroborated by our experimental limit. Moreover, tagging the isomeric state of $^{77\textrm{m}}$As can be utilised to further suppress the $^{77}$Ge background. Considering the similar experimental configurations of LEGEND-1000 and GERDA, the cosmogenic background in LEGEND-1000 at LNGS is estimated to remain at a sub-dominant level.

## Introduction

In-situ production of radioactive isotopes by atmospheric muons can represent a non-negligible background for experiments searching for rare events even when located deep underground. One such experiment is the GERDA (Germanium Detector Array) experiment [1] that searched for the neutrinoless double-beta ($0\nu \beta \beta$) decay in $^{76}$Ge located underground below a rock overburden of about 3.5 km.w.e. at the LNGS (Laboratori Nazionali del Gran Sasso) of INFN.

In Phase II of the GERDA experiment, 40 (after upgrade 41) high-purity germanium (HPGe) detectors made from material isotopically enriched in $^{76}$Ge were operated as bare crystals in a cryostat filled with 64 m$^3$ liquid argon (LAr). The LAr served as both a coolant and an instrumented active shield. It allowed effective detection of the argon scintillation light produced by background events that deposit energy in the argon surrounding the germanium detectors. The LAr cryostat was immersed in a 590 m$^3$ water tank equipped with photomultipliers which, together with scintillator plates on top of the setup, served as a further shield against external radiation and as a muon veto system. In the event of a trigger in one of the HPGe detector channels, all HPGe readout channels were recorded for off-line analysis. $0\nu \beta \beta$ decay candidate events were required to have a point-like energy deposition in a single HPGe detector, and no signal in the liquid argon or the muon system. Based on these selection criteria, a quasi-background free search was performed with a total exposure in detector mass accumulated over Phase II of 103.7 kg$\cdot$yr and, after combination with Phase I, a limit of on the half-life of $0\nu \beta \beta$ decay in $^{76}$Ge was set to $T_{1/2} > 1.8 \times 10^{26}$ at 90% C.L. [2].

Previous simulations [3] identified the delayed decays of $^{77}$Ge and its isomeric state $^{77\textrm{m}}$Ge, both produced by neutron capture on $^{76}$Ge, as the dominant cosmogenic backgrounds in GERDA. The Q$_\beta$ value of $^{77}$Ge (${2703}\,\hbox {keV}$) and of the isomeric state are both above the Q$_{\beta \beta }$ value of $^{76}$Ge (2039 keV). Thus, their $\beta$ decays can deposit energy in the region of interest at Q$_{\beta \beta }$ and mimic a signal-like event. Conversely, $^{77}$As, the decay product of $^{77}$Ge, does not contribute to the background, since with $Q_\beta = {684}\,\hbox {keV}$ it cannot contribute in the region of interest.

Recently, a $^{77\mathrm {(m)}}$Ge^1^ production rate of (0.21 $\pm 0.01 \text {(stat)}$ $\pm 0.07$ (sys)) nuc/(kg · yr) was obtained using a full GEANT4 simulation [4]. The systematic uncertainties were ${35}{\%}$, dominated by muon-induced neutron production and propagation. At this rate, the $^{77\mathrm {(m)}}$Ge background contribution at Q$_{\beta \beta }$ is estimated to be $\sim 10^{-5}$ cts/(keV$\cdot$ kg$\cdot$ yr) after applying the standard cuts used in GERDA. With the addition of a delayed coincidence cut as defined in the above paper, a background contribution of $(2.7\pm 0.3) \times 10^{-6}$ cts/(keV$\cdot$ kg$\cdot$ yr) can be achieved. Using this as a proxy for the planned $^{76}$Ge experiment LEGEND-1000 at LNGS this showed that the $^{77\mathrm {(m)}}$Ge background contribution can be suppressed low enough to achieve a background at Q$_{\beta \beta }$ of $<10^{-5}$ cts/(keV$\cdot$ kg$\cdot$ yr), which is a factor of 50 reduction with respect to GERDA [5]. Experimental validation of the predicted $^{77\mathrm {(m)}}$Ge production rate and constraining uncertainties using GERDA data are therefore of paramount importance to consolidate the background model of LEGEND-1000.

This paper summarizes the present analysis to quantify the in-situ production of $$^{77}$$Ge in its ground state ($$T_{1/2} = 11.21$$ h [7]) in GERDA by searching for its characteristic decay through the isomeric state ($$9/2^+ $$, 475 keV, $${T_{1/2}} = 114 \, \mu $$s) of its progeny $$^{77}$$As. The analysis uses the full data set of the GERDA Phase II experiment with an exposure of 103.7 kg$$\cdot $$yr. With the production rate estimate from above, this gives an expected number of about $$(22\pm 7)$$ $$^{77\text {(m)}}$$Ge nuclei in either its ground state or isomeric state.

**Fig. 1:**
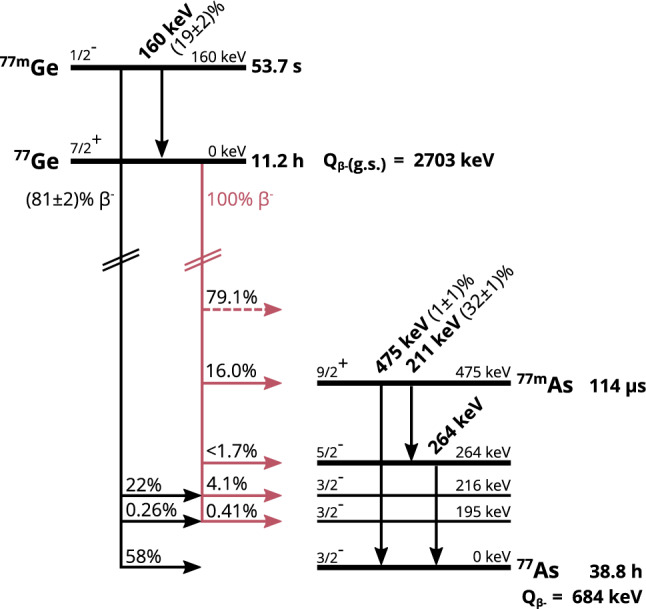
Simplified decay scheme of $^{77}$Ge and $^{77\textrm{m}}$Ge into $^{77}$As. $^{77\textrm{m}}$Ge only populates states $\le {216}\,\hbox {keV}$ while $^{77}$Ge also populates higher states including the $9/2^+$ isomeric state in $^{77}$As with ${T_{1/2}}={114}{\mu }$s and an excitation energy of ${475}\,\hbox {keV}$. Approximately ${16}{\%}$ of $^{77}$Ge decays into this isomeric state, while more than ${79}{\%}$ of $^{77}$Ge decays populate states above (not drawn) and can also populate the isomeric state from above. This plot was generated from the values of [7]

To isolate the $^{77}$Ge decays, we search for a coincidence between the prompt $\beta$ decay of $^{77}$Ge and the delayed de-excitation of the isomeric $^{77\textrm{m}}$As state. We require that the prompt energy deposition and the delayed de-excitation occur in the same detector. Figure 1 displays a simplified decay scheme of the $^{77}$Ge-$^{77}$As system. The time correlated signature of the beta decay into $^{77\textrm{m}}$As and its delayed de-excitation leads to so-called pile-up signals in the GERDA data stream.

Special processing of the GERDA data was performed to isolate time-correlated candidates since they were discarded in the standard GERDA $0\nu \beta \beta$ decay analysis. Furthermore, new digital signal processing (DSP) routines were developed to extract the physical parameters of the pile-up signal. The DSP routines have been validated using generated data and the candidate event selection efficiencies and the uncertainties in the energy reconstruction were determined.

A similar analysis searching for the production of $^{77}$Ge using the delayed de-excitation of the isomeric $^{77\textrm{m}}$As state was recently performed on the MAJORANA Demonstrator experiment data [6]. Considering the larger overburden of the Sanford Underground Research Facility (SURF) at 4.3 km w.e. compared to LNGS at 3.5 km w.e., the production rate of $^{77}$Ge is expected to be lower in comparison. This analysis found no candidate event for this mechanism, but for other cosmogenic isotopes it found a factor of 2 agreement between simulation and data. However, due to the different shielding material, i.e. lead in MAJORANA and LAr in GERDA, it is difficult to use these results to validate the GERDA simulation, which further motivates the work presented in this paper.

## Analysis procedure

We distinguish two classes of transitions: *prompt* transitions ($\beta$ and subsequent prompt $\gamma$ de-excitations), shown in Fig. 1, start from the ground state of $^{77}$Ge and end on the 475 keV (${T_{1/2}} = 114 \, \mu$s) isomeric state of $^{77\textrm{m}}$As. *Delayed* transitions, are those electromagnetic de-excitations that start from the 475 keV isomeric state and terminate at the ground state of $^{77}$As. Since the half-life of $^{77\textrm{m}}$As is longer than the charge collection time in GERDA’s high-purity germanium detector (HPGe) ($\lesssim {1.5}\,{\upmu }$s), the delayed transitions occur with sufficiently large time differences with respect to the prompt so that both transitions can be separated unambiguously in time. We call their combined occurrence in the same detector a *delayed coincidence*.

In Sect. 2.1 we identify the expected signature of the delayed coincidence in the GERDA experiment. Then, in Sect. 2.2 we present the DSP to reconstruct pile-up signals in the GERDA data and how we estimate their energies. Finally, in Sect. 2.3 we present the selection criteria to identify candidate delayed coincidences in the GERDA data and calculate the total selection efficiency.

### Signature in GERDA

In-situ cosmogenic interactions can produce both $^{77}$Ge and $^{77\textrm{m}}$Ge. The isomeric state $^{77\textrm{m}}$Ge undergoes an internal transition to the ground state $^{77}$Ge with $(19\pm 2)\%$. When $^{77\textrm{m}}$Ge decays directly to $^{77}$As, it has a ${99}{\%}$ probability of populating one of the four states that are energetically below the isomeric state $^{77\textrm{m}}$As. Therefore, $^{77\textrm{m}}$Ge decays cannot be tagged through the $114 \, \upmu$s delayed coincidence. Conversely, $(33\pm 1)\%$ of $^{77}$Ge decays populate the isomeric state $^{77\textrm{m}}$As (see Fig. 1). About half of the prompt decays ($(16\pm 1)\%$) that populate the isomeric state are direct transitions, while the other half come from consecutive gammas de-excitations from higher levels of $^{77}$As. The end-point energy of these decays into the isomeric state is ${2228}\,\hbox {keV}$ (${2703}\,\hbox {keV}$-${475}\,\hbox {keV}$). The rest of the beta decay branches (67%) populate other states that miss the isomeric state in the consecutive gamma decay.

While the beta particle will deposit its energy in the germanium detector where the $^{77\mathrm {(m)}}$Ge is produced, the gammas can also escape and deposit their energy in the liquid argon (LAr) or in other detectors. To simulate the prompt transitions of the $^{77}$Ge decay into $^{77\textrm{m}}$As in the GERDA Phase II experiment, we used the MaGe simulation framework [8]. We define the multiplicity as the total number of detectors with coincident energy deposition above ${40}\,\hbox {keV}$. At least one of these detectors must have an energy deposition above ${200}\,\hbox {keV}$. This additional condition avoids systematic uncertainties in modeling the online trigger threshold, which had different values during different data acquisition periods but was always set well below the above value. We find that $>75\%$ of prompt transitions occur with a multiplicity of one, i.e., all energy is deposited in a single detector.

**Fig. 2:**
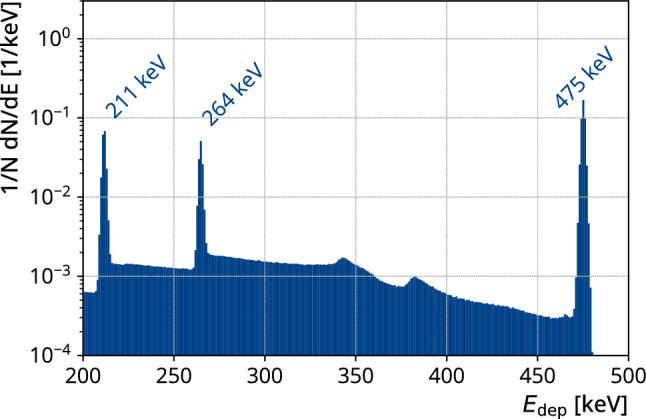
Simulated energy distribution of the delayed gamma emission from the isomeric state in $^{77}$As deposited in a HPGe. The peaks correspond to the full energy deposition of both gammas in the detector at ${475}\,\hbox {keV}$ or the full energy deposition of only one of the gammas in the detector at ${211}\,\hbox {keV}$ or ${264}\,\hbox {keV}$. The energy resolutions of each individual detector derived from the standard GERDA analysis were implemented

The delayed transitions were also simulated with MaGe. We found that ${65}{\%}$ of the energy is deposited only in the same detector as the prompt transition. Figure 2 shows the energy deposited in an HPGe of a delayed transition with multiplicity one. The spectrum shows full energy peaks at ${211}\,\hbox {keV}$, ${264}\,\hbox {keV}$, and at ${475}\,\hbox {keV}$ due to the summation of the two gammas or the single gamma transition (see Fig. 1).

**Fig. 3:**
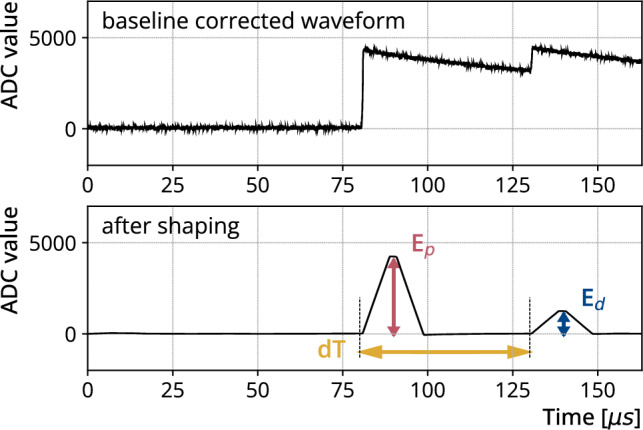
An example of pile-up signal reconstruction. (Top) Example of a pile-up event waveform in the GERDA data stream. (Bottom) The waveform of the example pile-up event after applying a trapezoidal filter. The time difference between the signals is estimated by taking the difference between the triggers. Finally, the signal heights are extracted with a fixed time pick off

GERDA used charge-sensitive preamplifiers with RC-feedback that shape the physical signal from a HPGe detector into a pulse consisting of a rapid change in output voltage followed by a slow exponential decay ($\tau \sim 150\;\upmu$s) back to the baseline. The time difference between the prompt and delayed transitions is of the same order as the decay constant of the charge sensitive amplifier. Therefore, in the previously mentioned most likely case where the delayed transition only deposits energy in the same detector, the signal produced by the delayed transition will appear as a pulse that lies on top of the pulse produced by the prompt transition also known as pile-up (e.g., see Fig. 3, top).

An energy deposition in any of the 40(41) HPGe detectors generated a synchronous readout of the waveforms of all HPGe detectors. These coincident waveforms are referred to as one event. The length of the waveforms is ${164}\,{\upmu }$s, with the initial trigger centered at about ${80}\,{\upmu }$s Therefore, a pile-up signal can occur either in the same waveform as the prompt signal (as see the example Fig. 3) or in a waveform of a subsequent event. In the GERDA experiment, the data acquisition (DAQ) system is configured to record a new event if a trigger occurs more than ${50}\,{\upmu }$s after the previous one, with the second waveform centered at ${80}\,{\upmu }$s. If the interval between two triggers is shorter than the full waveform duration (${164}\,{\upmu }$s), the corresponding waveforms will overlap, meaning they share a common set of samples. Since the standard GERDA analysis rejects overlapping waveforms [9], we have implemented a new DSP tool to accurately reconstruct pile-up signals also for overlapping waveforms.

**Fig. 4:**
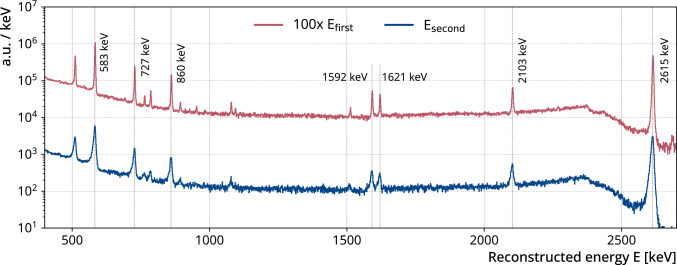
Reconstructed energy spectra for the first and second pulse in a pile-up signal in the calibration data where two signals are contained in the waveform of one event. The red histogram corresponds to the first pulses energies, while the blue histogram corresponds to the second pulses energies. The former distribution was scaled by a factor of 100 to illustrate the differences between the spectra. Both spectra reconstruct the expected $^{208}$Tl gamma lines well. The spectra of the second pulses consistently shows larger resolution and a slight tail to lower energies

### Pile-up signal reconstruction

The new DSP is based on the validated MAJORANA GERDA Data Objects Library (MGDO), which are also used in the standard GERDA DSP [10]. The DSP transforms the original waveform with trapezoidal filters consisting of a pole-zero correction, a moving window differentiation, and a moving window average filter. A selection of filter lengths was chosen that are optimized for energy resolution and detection sensitivity. The filter length for a given pile-up signal is selected in steps according to the minimum time difference between the triggers. For example, for time differences ${>18}\,{\mu }$s, the moving window differentiation is ${10}\,{\mu }$s wide and the moving window average is ${8}\,{\mu }$s, resulting in a total filter length of ${18}\,{\mu }$s and a flat top of ${2}\,{\mu }$s. The time difference between signals in the same waveform is calculated as the difference between trigger positions. For pile-up signals in different events we extract the time difference using their timestamp in ns. The height of the signals are extracted with a fixed time pick-off at the ${75}{\%}$ point on the flat top. Figure 3 bottom shows an example of the DSP, extracting the peak heights and time difference between the signals. The DSP also extracts additional quality parameters from the waveforms such as a negative trigger to reject electromagnetic noise and non-physical pulses. We ran the new DSP on waveforms containing two pulses, extracting the heights of both the prompt and the delayed event, and on waveforms containing only the delayed pulse. For all other situations, we found that the original DSP gave similarly good results, and we used its energy estimation.

To calibrate the signal heights extracted from the waveform with the new DSP, we use the waveforms of signals with already estimated energies from the standard GERDA analysis. We apply the same trapezoidal filter to 50 consecutive single signal waveforms after the delayed coincidence candidate in the same detector. We plot their signal height against their previously estimated energy, associate them with an uncertainty corresponding to the resolution of the detector at that energy and extract the calibration by fitting the plot with a linear function. This gives us the calibrated reconstructed prompt ($E_\textrm{p}$) and delayed ($E_\textrm{d}$) energies.

Since this is a non-standard approach to energy calibration, we performed several cross-checks. In GERDA, to obtain a calibration, dedicated calibration runs were performed before and after each physics run [11]. During those, three $^{228}$Th sources were automatically lowered into the LAr cryostat in close proximity to the HPGe detectors. The calibration data are populated with a large contribution of pile-ups from the gammas emitted by the progeny $^{208}$Tl. Since these signals are not correlated, they are called first and second instead of prompt and delayed pulse. Figure 4 shows the energy distribution of the first and second pulse energies of pile-up signals in the calibration data, where the two signals are contained in the waveform of one event. We can reconstruct the expected gamma lines for both pulses in a pile-up signal. The energy distribution of the first pulse shows energy resolutions of e.g. a Full Width Half Maximum (FWHM) of 4.2 keV at 2.6 MeV. Compared to the exposure weighted resolution in the standard GERDA analysis of about 3.7 keV at 2.6 MeV this value is slightly higher. In contrast to the standard GERDA analysis, the DSP parameters in this study were optimized for pile-up recognition rather than for achieving optimal energy resolution. The energy distribution of the second pulse exhibits a significant degradation in resolution. For instance, a FWHM of 8.4 keV at 2.6 MeV is roughly twice that of the first pulse. This degradation arises because the second pulse occurs on the falling tail of the first pulse, resulting in suboptimal baseline reconstruction. In addition, the gamma lines exhibit low-energy tailing and a slight energy shift of approximately ${-1}$ keV at 2.6 MeV, here referred to as the energy bias (see Fig. 4).

To further investigate the energy resolution and bias of individual candidates delayed coincidences, we generated data with similar signatures. We chose single pulse waveforms from the calibration data with similar prompt energy to the candidate prompt pulse. Then we selected another single pulse waveform with energies near the $^{208}$Tl gamma lines as the delayed pulse and added them with a time difference similar to the candidate delayed coincidences. Subsequently, we reconstructed the energy of the generated pile-up signal with the DSP and extracted the energy resolution and bias from the reconstructed gamma lines by fitting the peaks with a Gaussian peak, a linear background, a step function, and a tail function similar to [11]. We also modeled the energy resolution in a similar way by taking the square root of a linear function and fitting it to the peaks full-width-at-tenth-maximum (FWTM). To obtain the energy resolution of the candidate pile-up signal, we interpolated this model at the reconstructed delayed energy. We defined the bias of the candidate pile-up signal energy as the largest difference between the reconstructed peak mean energy and the expected energy of all gamma lines. We found that the bias is the largest for delayed coincidences with short time differences but usually well below ${1}\,\hbox {keV}$. With this we estimated a FWTM and bias range for each candidate delayed coincidence.

### Candidate selection

Based on the simulation results, we define the selection criteria for the prompt and delayed energy and the multiplicity as well as the time difference of the candidates.

**Table 1:** Delayed coincidence selection efficiencies. The uncertainty of these values is in the order of ${<0.5}{\%}$

Energy and multiplicity selection efficiency $\epsilon _{\textrm{em}}$	$34.5\%$
Prompt contribution $\epsilon _{\textrm{em}}^\textrm{p}$	$74.6\%$
Delayed contribution $\epsilon _{\textrm{em}}^\textrm{d}$	$46.2\%$
Time difference selection efficiency $\epsilon _{\textrm{dT}}$	$99.3\%$
Pile-up signal selection efficiency $\epsilon _{\mathrm {pile-up}}$	$94.9\%$
Total selection efficiency $\epsilon _{\textrm{total}}$	$32.4\%$

We define a selection condition for prompt transition candidates by requiring that they have (i) a multiplicity of one and (ii) an energy $E_\textrm{p}$ between ${200}\,\hbox {keV}$ and ${2228}\,\hbox {keV}$ equal to the maximum total deposited energy. The simulation shows that ${74.6}{\%}$ of prompt transitions satisfy these conditions.

We further define a delayed transition selection condition that candidate delayed transitions must (i) have multiplicity one, (ii) occur in the same detector as the prompt transition, and (iii) have one of the three gamma energies (${211}\,\hbox {keV}$, ${264}\,\hbox {keV}$ or ${475}\,\hbox {keV}$) lie within the respective reconstructed energy acceptance region $E_\textrm{d}$ defined by the FWTM range plus bias. We chose to use the FWTM (containing ${96}{\%}$ of the peak area) rather than the FWHM to account for possible tails as gamma lines in the energy spectrum of the delayed candidate signals may deviate from the expected Gaussian distribution (see Fig. 4). To account for a potential bias in the reconstructed delayed energies, we extend the acceptance region of an individual delayed coincidence candidate asymmetrically by the minimum and maximum bias described in Sect. 2.2. We find that ${46.2}{\%}$ of the delayed transitions satisfy the above conditions. The energy and multiplicity selection efficiency of ${34.5}{\%}$ in Table 1 is the combined efficiency to select both the prompt and the delayed transition with these conditions.

Finally, we require that the time difference *dT* between a prompt and a delayed transition candidate be no greater than five times the lifetime of the isomeric state (${822}\,{\upmu }$s). The corresponding time difference selection efficiency is also given in Table 1.

We now define the criteria for selecting the candidates based on the quality parameters extracted from the standard GERDA analysis as well as the pile-up reconstruction using our DSP. Due to the difference in signature between delayed coincidences with signals in the same or different waveforms, we define three different regions depending on the time difference between the signals.

1. For time differences less than ${70}\,{\upmu }$s, the two signals will be contained in one waveform. This is because the first signal is approximately in the center at ${80}\,{\upmu }$s of the long ${164}\,{\upmu }$s waveform. We require that the quality parameters that are extracted from the new DSP satisfy a set of conditions. A candidate for a delayed coincidence with corresponding time differences must have (i) exactly two triggers and (ii) pass through the additional quality parameter cuts.
2. For time differences between ${70}\,{\upmu }$s and ${164}\,{\upmu }$s, the two signals are contained in separate events, but are still so close together that their waveforms overlap. Since the DAQ records a new event when a trigger occurs at least ${50}\,{\upmu }$s after the previous one, we are sensitive to all signals in that time range. In the standard GERDA analysis, the second event was discarded before the DSP step. This is due to a short busy period of the DAQ of the order of 100 ns during the saving process of the first event, which interrupted the recording of the waveform of the following event. We reconstructed such waveforms by interpolating in these periods. Since these events were not processed in the standard GERDA analysis, we applied DSP to all its waveforms. Using the extracted quality parameters, we require that a candidate delayed signal waveform with corresponding time difference (i) contains only one trigger and (ii) pass through the additional quality parameter cuts.
3. For time differences above ${164}\,{\upmu }$s, the waveforms of the two events no longer overlap, and both events have been processed in the standard GERDA analysis. We use the standard GERDA quality conditions to differentiate signal candidates from non-physical signals. We require that the prompt waveform satisfy all standard GERDA quality conditions, while the delayed waveform satisfies a subset of these conditions consistent with a signal on an exponential tail. For these events, we did not apply the new DSP to either the prompt or the delayed waveform because the standard GERDA analysis already provided reliable values of the prompt and delayed energies.

**Fig. 5:**
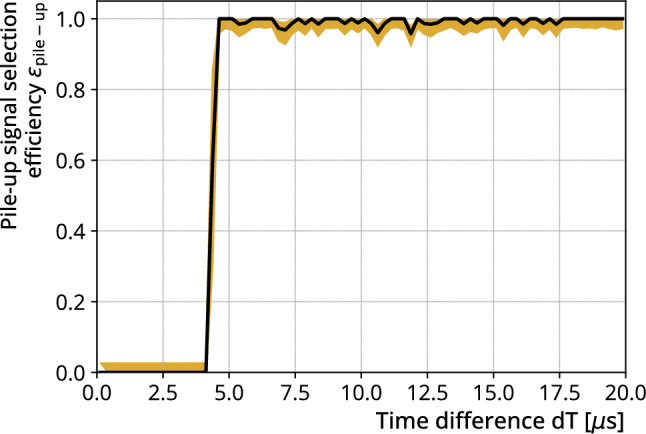
Pile-up signal selection efficiency plotted over the time difference between generated pile-up signals. Our new DSP is sensitive for delayed coincidences starting at time differences ${>4.5}\,{\upmu }$s. Individual events in this range were rejected due to quality cuts. Black: central value. Yellow: 68% uncertainty band

To estimate the resulting pile-up signal selection efficiency, we generated pile-up signals with energies in the corresponding energy ranges and time differences sampled from an exponential distribution according to the lifetime of $^{77\textrm{m}}$As. We define the pile-up signal selection efficiency as the number of signals that pass the entire pile-up signal selection procedure over the total amount generated. The final pile-up signal selection efficiency is given in Table 1. We tested whether the pile-up signal selection efficiency depends on certain parameters. Figure 5 shows the pile-up signal selection efficiency plotted over the time difference range up to ${20}\,{\upmu }$s. It shows a jump from zero to almost one at ${4.5}\,{\upmu }$s, which shows that our analysis is sensitive to pile-ups with time differences above this threshold. The efficiency of pile-up signal selection remains constant above ${20}\,{\upmu }$s up to the upper limit of time difference selection at $5\,\tau _{77\textrm{m}}\,\,\textrm{As} = {822}\,{\upmu }$s. Therefore, the only dead time in our analysis is due to the delayed signals that occur at time differences smaller than ${4.5}\,{\upmu }$s. We also tested for a prompt or delayed energy dependence and found it to be constant over the ranges of interest.

The total selection efficiency $\epsilon _{\textrm{total}} = \epsilon _{\textrm{em}} \times \epsilon _{\textrm{dT}} \times \epsilon _{\mathrm {pile-up}}$ amounts to $32.4\%$.

**Fig. 6:**
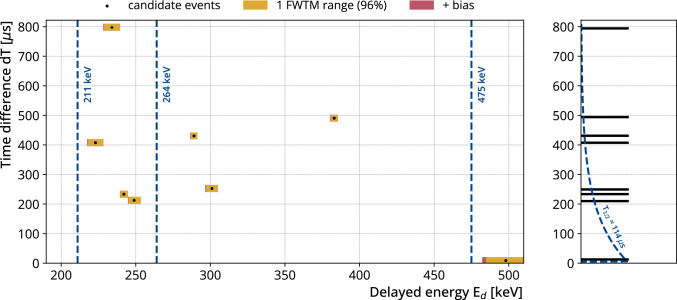
The distribution of all 8 candidate delayed coincidences after the multiplicity condition plotted *dT* over $E_\textrm{d}$. The energy windows around the points correspond to the linear combination of the full-width-at-tenth-maximum (FWTM) window (yellow) plus the asymmetric bias window (red). The FWTM window covers about $97\%$ of the gamma peak area. The vertical size of the data is enlarged for better visualization. The blue lines correspond to the gamma energies of the internal transitions from $^{77\textrm{m}}$As . A delayed coincidence candidate is rejected, if its energy window misses any of the three gamma lines. We found no candidate delayed coincidence that satisfies this condition resulting in $N_\textrm{obs} = {0}\,\hbox {cts}$. The right part shows a projection of the candidates onto *dT*. The blue line corresponds to the expected distribution for $^{77\textrm{m}}$As delayed coincidences

## Result

We applied the pile-up signal selection to the entire GERDA Phase II data set, which has an exposure of 103.7 kg$\cdot$yr. Figure 6 shows a scatter plot of the delayed coincidence candidates that pass all the selection criteria of the previous section apart from the delayed energy selection. The black dots represent the reconstructed values of delayed energy and time difference, while the colored bars in x-direction represent the delayed energy FWTM plus bias acceptance region (linearly summed) estimated individually for each candidate. A candidate is accepted if its reconstructed delayed energy acceptance region overlaps with one of the expected gamma energies. We find that there is no candidate delayed coincidence that passes this selection ($N_\textrm{obs} = {0}\,\hbox {cts}$).

Random coincidences are the only known background source in this analysis. Using the average signal rates in each detector for the 1 FWTM energy ranges around the gamma lines, we calculated the number of expected random coincidence signals considering our selection criteria to be $N_\textrm{rc} = {0.04}\,\hbox {cts}$.

For a wider range of delayed energies between ${200}\,\hbox {keV}$ and ${500}\,\hbox {keV}$ the expected number of random coincidence signals is ${0.2}\,\hbox {cts}$ compared to eight delayed coincidence candidates observed (see Fig. 6). The time and energy distributions of these signals differ significantly from the expected $^{77}$Ge delayed coincidence time and energy distributions, and thus can be excluded as a majority contributor to these events. If we ignore the time distribution, for the $^{77}$Ge delayed coincidences that deposit energy above ${200}\,\hbox {keV}$, the fraction of energy depositions in the continuum region is around ${26}{\%}$, with the rest in the peaks. Without an event in the peaks, it is therefore feasible that one or two events in the continuum could be $^{77}$Ge delayed coincidences, but more would be unlikely. A search for other sources generating delayed coincidences such as delayed neutron capture in muon-induced showers, $^{214}$Bi-$^{214}$Po decays, or delayed de-excitation in other isotopes, was performed but was inconclusive.

With $N_\textrm{obs} = {0}\,\hbox {cts}$ and $N_\textrm{rc} = {0.04}\,\hbox {cts}$, we can determine an upper limit of $<{2.4}\,\hbox {cts}$ ($90\%$ CL) on the number of delayed coincidences by applying Feldman–Cousins method [12]. We can convert the number of delayed coincidences to the number of $^{77}$Ge decays by dividing it with the product of the total selection efficiency and the ratio of delayed coincidences per $^{77}$Ge decay ($(33\pm 1)\%$, see Fig. 1). We treat the uncertainty by integrating over the nuisance parameter as described in [13, 14]. We found an upper limit on the number of $^{77}$Ge decays of $N_{{77}\textrm{Ge}} = {22.4}\,\hbox {decays}$ ($90\%$ CL).

Considering that the $^{77}$Ge has a half-life much shorter than the time during which GERDA Phase II acquired the 103.7 kg$\cdot$yr exposure, any $^{77}$Ge must have been produced in-situ. We can therefore calculate an upper limit on the $^{77}$Ge production rate of $<{0.216}$ nuc/(kg$\cdot$ yr) ($90\%$ CL).

The $^{77}$Ge in GERDA is mainly produced by cosmogenic activation. Radiogenic neutrons from natural decay chains originating outside the experiment are absorbed by the water tank and cannot reach the HPGe detectors. In the LAr cryostat, a cosmic muon can produce particle showers with high neutron multiplicity. The vast majority of neutrons are captured by $^{40}$Ar or by the water in the water tank. A minority is captured by $^{76}$Ge, producing either the ground state $^{77}$Ge or the isomeric state $^{77\textrm{m}}$Ge. The production ratio between the two depends on the kinetic energy of the neutron when absorbed. Following the arguments from our previous work [4] and corroborated by statistical model calculations [15], we assign a probability for the direct ground state population of $\epsilon _{d} = (50\pm 10)\%$. In addition, the probability of the internal transition from the isomeric state into the ground state of $\epsilon _\textrm{IT} = (19\pm 2)\%$ has to be taken into account. Therefore, the probability to populate the ground state $\epsilon _\textrm{g}$ after neutron capture is

$$\epsilon _\textrm{g} = (\epsilon _\textrm{d} + (1 - \epsilon _\textrm{d}) \cdot \epsilon _\textrm{IT}) = (59.5\pm 8.1)\%$$.

Assuming that all delayed coincidences are of cosmogenic origin, we can use $\epsilon _\textrm{g}$ to calculate the sum of the $^{77}$Ge and $^{77\textrm{m}}$Ge production rate. We treat the nuisance parameters as before. Applying the Feldman–Cousins method, we get a total $^{77\mathrm {(m)}}$Ge production rate of

$< {0.38}$  nuc/(kg$\cdot$ yr) ($90\%$ CL).

This is the strongest experimental constraint on the $^{77(\textrm{m})}$Ge production rate at LNGS and is about a factor of two larger than the MC prediction of $0.21 \pm 0.01 \text {(stat)} \pm 0.07 \text {(sys)}$  nuc/(kg$\cdot$ yr). For completeness, we refer here to a previous analysis performed on the GERDA data, in which an upper limit of $<4.1$ nuc/(kg$\cdot$ yr) ($90\%$ CL) was derived in [16].

**Fig. 7:**
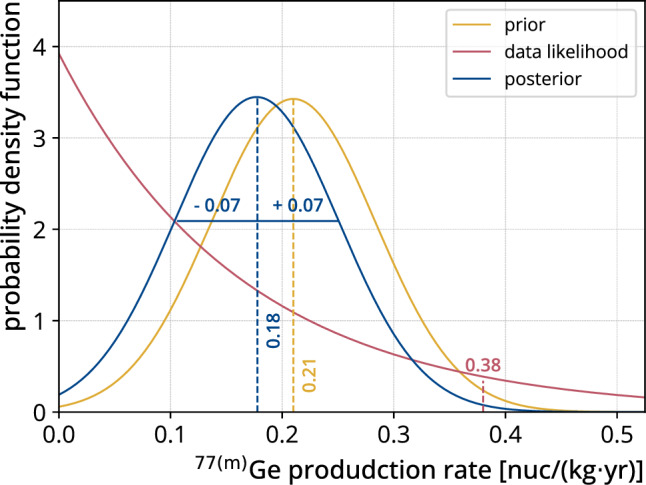
Bayesian update of the simulated production rate using the likelihood of the GERDA data estimated in this analysis

With our constraint on the $^{77\mathrm {(m)}}$Ge production rate, we can update the predictions from previous simulations [4]. To this end, the simulated production rate estimate is treated as a prior with a Gaussian distribution centered at 0.21 nuc/(kg$\cdot$ yr) and an uncertainty of 0.07  nuc/(kg$\cdot$ yr) ($1 \sigma$). Our constraint on the $^{77\mathrm {(m)}}$Ge production rate is modeled by an exponential function as illustrated in Fig. 7. The posterior has a central value of 0.18 nuc/(kg$\cdot$ yr) with a $1\sigma$ credibility interval of [0.106, 0.251] nuc/(kg$\cdot$ yr). Relatively, the posterior predicts the simulated production rate scales by a factor of $0.85^{+0.35}_{-0.34}$ compared to the original estimate.

Previous simulations of the background from $^{77\mathrm {(m)}}$Ge decays in GERDA predicted a background index (BI) of $(2.7\pm 0.3) \times 10^{-6}$ cts/(keV$\cdot$ kg$\cdot$ yr) with individual contributions of $(1.2\pm 0.5) \times 10^{-6}$ cts/(keV$\cdot$ kg$\cdot$ yr) and $(1.5\pm 0.2) \times 10^{-6}$ cts/(keV$\cdot$ kg$\cdot$ yr) from $^{77\textrm{m}}$Ge and $^{77}$Ge respectively excluding the previously mentioned systematic uncertainties of ${35}{\%}$ [4]. This BI is achieved after active background suppression (i.e. detector anti-coincidence, rejection by liquid argon anti-coincidence and pulse shape discrimination (PSD)), and after applying a veto condition after tagged muons. Applying the scaling factor for the $^{77\mathrm {(m)}}$Ge production rate, we estimate a contribution to BI of $(2.3\pm 0.1\text {(stat)}\pm 1.0\text {(sys)}) \times 10^{-6}$ cts/(keV$\cdot$ kg$\cdot$ yr). The statistical uncertainty is due to the finite exposure simulated in the previous simulation study. The systematic uncertainty consists of the approximately symmetric distribution of the production rate scaling factor with standard deviation ${35}{\%}$ and the uncertainty of the direct population of the ground state after production of ${10}{\%}$.

Tagging the delayed coincidence decay through the isomeric state in $^{77}$As can not only be used to estimate the production rate, but also to tag and reject $^{77}$Ge decays in the $0\nu \beta \beta$ decay search. To estimate how many such $^{77}$Ge decays can be additionally rejected, we simulated $^{77}$Ge decays using a MaGe simulation of the GERDA setup to and modeled the active background suppression in the same way as in [4]. We found that of the $^{77}$Ge decays with an energy around Q$_{\beta \beta }$, ${92}{\%}$ are rejected by the active background suppression. Of the surviving events, about $(62\pm 1)\%$ produce a delayed coincidence signal with E$_\textrm{d} > {200}\,\hbox {keV}$ and $dT > {10}\,{\upmu }$s. The large contribution of such events can be explained by the fact that ${16}{\%}$ of $^{77}$Ge decay directly into the isomeric state, which are simple beta decays with a similar topology to $0\nu \beta \beta$ decays and therefore predominantly survive the analysis cuts. By rejecting events with the delayed coincidence signal mentioned above, we estimate that a $^{77\mathrm {(m)}}$Ge background index contribution of $(1.50\pm 0.07\text {(stat)}\pm 0.67\text {(sys)}) \times 10^{-6}$ cts/(keV$\cdot$ kg$\cdot$ yr) is achievable. The contribution of this rejection to the $0\nu \beta \beta$ decay survival fraction is negligible due to the low random coincidence rate.

## Implications and conclusions

LEGEND is the successor experiment to GERDA, which in its first phase, LEGEND-200, reuses the GERDA cryostat at LNGS and operates up to 200 kg of HPGe detectors enriched in the isotope $^{76}$Ge. In the second phase, LEGEND-1000, one tonne of enriched HPGe detectors will be deployed in a new experimental infrastructure in Hall C of LNGS. After one year of LEGEND-200 data taking with the final detector mass, the sensitivity of the analysis presented here will double, tightening the constraint on the $^{77\mathrm {(m)}}$Ge production rate by a factor of two, or potentially revealing a signal.

A previous simulation for LEGEND-1000, also based on GEANT4, gave a $^{77\mathrm {(m)}}$Ge production rate of $0.33 \pm 0.01 \text {(stat)} \pm 0.12 \text {(sys)}$ nuc/(kg$\cdot$ yr) [17]. The approximately 1.6-fold increase in the $^{77\mathrm {(m)}}$Ge production rate is attributed to the larger LAr volume compared to GERDA, which results in a higher neutron flux at the HPGe detectors and consequently a greater neutron capture rate. As described in [17], a neutron moderator will be installed around the HPGe strings, dividing the liquid argon (LAr) volume into an inner and an outer region. This design aims to moderate neutrons crossing from the outer to the inner region to lower energies where capture on $^{40}$Ar is more likely than on $^{76}$Ge. By thus removing the contribution of neutrons originating outside the moderator, the $^{77\mathrm {(m)}}$Ge production rate is effectively reduced to a level below that observed in GERDA. As this decouples the production rate from the size of the cryostat, changes in the ongoing development of the experiment geometry are not expected to change this estimate. Furthermore, study [17] estimates that only ${2.3}{\%}$ of all $^{77\textrm{m}}$Ge decays in LEGEND-1000 will survive the veto condition following tagged muons. This 2.6-fold reduction in the survival fraction, compared to the ${6}{\%}$ observed in GERDA [4], is primarily attributed to the tagging of neutrons in liquid argon in hadronic showers, which exhibit high neutron multiplicity. Combined with the tagging of the $^{77}$Ge decays transitioning through the isomeric state in $^{77}$As, we estimate that the in-situ cosmogenic background contribution for LEGEND-1000 will be below $10^{-6}$ cts/(keV$\cdot$ kg$\cdot$ yr).^2^

The goal of LEGEND-1000 is to achieve a background index of $<10^{-5}$ cts/(keV$\cdot$kg$\cdot$yr) for a quasi-background-free search for $0\nu \beta \beta$ decays [5]. The contribution of in-situ cosmogenic background thus accounts for ${\le 10}{\%}$ of the total background budget. These findings indicate that the rock overburden at LNGS, combined with the suppression strategies discussed in [4, 17], and the delayed coincidence method to identify $^{77}$Ge decay via the isomeric state of $^{77}$As presented in this paper, are highly effective in reducing the cosmogenic background in LEGEND-1000 to a sub-dominant level.

## Data Availability

This manuscript has no associated data. [Authors’ comment: All relevant results are collected in Figs. 6 and 7. For further information contact the GERDA Collaboration (gerda-eb@mpi-hd.mpg.de).]

## Footnotes

1 The notation $^{77\mathrm {(m)}}$Ge represent both $^{77}$Ge and $^{77\text {m}}$Ge.

2 This estimate is based on the background estimates of GERDA for $^{77\textrm{m}}$Ge of $(1.2\pm 0.5) \times 10^{-6}$ cts/(keV$\cdot$ kg$\cdot$ yr) and for $^{77}$Ge of $(1.5\pm 0.2) \times 10^{-6}$ cts/(keV$\cdot$ kg$\cdot$ yr), and the survival fractions for the additional cuts (survival fraction of $^{77}$Ge after $^{77\textrm{m}}$As decay tagging: $(1-62\%) = 38\%$; survival fraction of $^{77\textrm{m}}$Ge after muon tagging: $2.3\%$ ($6\%$) in LEGEND-1000 (GERDA) after scaling by the Bayesian update of the simulation estimate (0.85).

$$\begin{aligned}  &   8.8 \times 10^{-7} \text {cts}/(\text {keV}\cdot \text {kg}\cdot \text {yr}) \\  &   \quad = 0.85 \times [ 1.5 \times 10^{-6} \text {cts}/(\text {keV}\cdot \text {kg}\cdot \text {yr}) \times 38\% \\  &   \qquad + 1.2 \times 10^{-6} \text {cts}/(\text {keV}\cdot \text {kg}\cdot \text {yr}) \times (2.3\% / 6\%) ] \end{aligned}$$

Since the background contribution of $^{77\textrm{m}}$Ge in GERDA already accounted for the survival fraction, the corresponding LEGEND-1000 estimate was scaled according to the better survival fraction. In addition, the background index contribution is expected to be lower in LEGEND-1000 compared to GERDA, as the neutron moderator will reduce the production rate of cosmogenic isotopes. These estimates can also be refined by use of the results from [15].

## Code Availability Statement

This manuscript has no associated code/software. [Authors’ comment: Code/Software sharing not applicable to this article as no code/software was generated or analysed during the present study.]
